# A quantization method based on threshold optimization for microarray short time series

**DOI:** 10.1186/1471-2105-6-S4-S11

**Published:** 2005-12-01

**Authors:** Barbara Di Camillo, Fatima Sanchez-Cabo, Gianna Toffolo, Sreekumaran K Nair, Zlatko Trajanoski, Claudio Cobelli

**Affiliations:** 1 Information Engineering Department, University of Padova, Padova, 35131 Italy; 2 Institute for Genomics and Bioinformatics and Christian Doppler Labor, Graz University of Technology, Graz, 8010 Austria; 3 Endocrinology Division, Mayo Clinic, Rochester, Minnesota 55905, USA

## Abstract

**Background:**

Reconstructing regulatory networks from gene expression profiles is a challenging problem of functional genomics. In microarray studies the number of samples is often very limited compared to the number of genes, thus the use of discrete data may help reducing the probability of finding random associations between genes.

**Results:**

A quantization method, based on a model of the experimental error and on a significance level able to compromise between false positive and false negative classifications, is presented, which can be used as a preliminary step in discrete reverse engineering methods. The method is tested on continuous synthetic data with two discrete reverse engineering methods: Reveal and Dynamic Bayesian Networks.

**Conclusion:**

The quantization method, evaluated in comparison with two standard methods, 5% threshold based on experimental error and rank sorting, improves the ability of Reveal and Dynamic Bayesian Networks to identify relations among genes.

## Background

A crucial issue in microarray studies is the elucidation of how genes change expression and interact as a consequence of external/internal stimuli such as drug assumption, hormone stimulation, illness, etc. Given a system whose elements regulate each other, inference of the regulatory network from the observed dynamics of the system is denoted as reverse engineering. Several approaches are available in the literature; among them Boolean models [1-4], models based on differential equations [5-7], Bayesian networks [8-11] and methods based on measurement of pair-wise gene expression correlation [12-16]. Application of reverse engineering to real data suffers from some drawbacks. First, although the regulatory network controlling gene expression involves RNAs, regulatory regions on DNA, proteins and metabolites, usually only gene expression data from microarray experiments are available and used as a proxy of protein activity. Therefore, gene-gene interactions identified using reverse engineering methods from microarray data are not, in general, direct regulatory actions or physical interactions, but functional relations [17]. Second, experiments are often characterized by a very limited number of samples with respect to number of analyzed genes (a data-poor situation unfortunately very common in practice, as evident searching Gene Expression Omnibus database [18] for time series experiments). This e.g. renders difficult to estimate with sufficient accuracy model parameters describing differential equations or, in case of Bayesian networks, conditional probability distributions. Application of reverse engineering thus results in an exceedingly large number of false positive interactions. In this situation, a more realistic objective is to focus on groups of genes whose expression profiles are linked to each other by a set of cause-effect relationships, rather than reconstructing the entire regulatory network underlying gene behaviour. To this purpose, the use of discrete approaches offers some advantages, since it may reduce the probability of finding random associations between genes and limits the dimensionality of the problem and thus the computational time needed to search the space of possible relationships between genes. However, discrete data offer a simplified representation of reality even if, as pointed out by Shmulevich et al. [3], there is evidence that meaningful biological information can be extracted from discrete gene expression data.

Here we explicitly address short time series microarray experiments and explore the use of a discrete approach to identify gene relationships. A quantization method is presented, using a threshold which is optimized based on a model of the experimental error and on a compromise between false positive and false negative classifications. Two standard quantization methods are also considered based, respectively, on a model of the experimental error but with a threshold corresponding to an arbitrary 5% significance level, and on rank sorting. The three methods are evaluated based on their ability to identify relations among genes when used as preliminary step to two discrete reverse engineering methods: Reveal [2] and Dynamic Bayesian Networks (DBN) [19]. The analysis is performed on synthetic data generated as continuous profiles from simulated regulatory networks consisting of different sub-networks with random scale-free topology. The ability to identify relations among genes belonging to the simulated sub-networks is used to quantify the performance of the methods.

## Methods

### Quantization

Expression of gene x at time t is quantized in three levels (-1,0,1), representing "underexpressed", "not differentially expressed" or "overexpressed" values with respect to baseline, according to the following rules:

x(t) is quantized as +1 if it exceeds its basal value x_b _by at least θ

(x(t) - x_b_) > θ ⇒ x(t) = +1     (1)

as -1 if x_b _exceeds x(t) by at least θ:

(x(t) - x_b_) < -θ ⇒ x(t) = -1     (2)

as 0 if x(t) differs from x_b _for less than θ:

|x(t) - x_b_| ≤ θ ⇒ x(t) = 0     (3)

The novelty of the method is how the threshold θ is fixed from the distribution of the error, on the basis of a compromise between false positives (incorrect ± 1) and false negative (incorrect 0) classifications. More in details, to derive θ a model is required for (x(t) - x_b_) distribution, under the null hypothesis that x(t) and x_b _are two realizations of the same variable. When dealing with real data, experimental replicates can be used to derive the null hypothesis distribution (see Appendix for details). θ is then evaluated according to a significance level α, but, rather than fixing it a priori, α is optimized so as to compromise between false positive and false negative classifications. The expected number of false positives (FP) is approximated as the product of α by the number N_0 _of not differentially expressed genes:

FP = N_0 _·α     (4)

The expected number of true negatives (TN) is then:

TN = N_0 _- FP = N_0 _·(1 - α)     (5)

Hence, the expected number of false negatives (FN) is derived by subtracting TN (Equation 5) and the sum of true positives and false positives (i.e. the number S_α _of ±1 classifications obtained using the significance level α) from the total number of genes N:

FN = N - TN - (TP + FP) = N - N_0 _·(1 - α) - S_α _    (6)

A compromise between FP (Equation 4) and FN (Equation 6) is achieved if the following condition holds:

FN = FP ⇔ N - N_0 _·(1 - α) - S_α _= N_0 _·α ⇔ N - N_0 _= S_α _    (7)

N_0 _is unknown and is estimated using the bootstrap based procedure described in [20]. S_α _is evaluated using Equations (1–3) for different values of α, and α that guarantees S_α _= N - N_0 _is then selected.

### Dynamic Bayesian Networks and Reveal

The performance of the quantization method is assessed on synthetic data, with two discrete reverse engineering algorithms: Dynamic Bayesian Networks (DBN) [19] and Reveal [2]. The two algorithms are implemented using a three steps procedure: 1) clustering of identical discrete profiles; 2) search for causal relationships using reverse engineering algorithms; 3) sub-network identification.

#### Step1: clustering

Genes with identical discrete profile are grouped together since identical profiles give the same information content. This step is useful to reduce the computational time since a smaller number of expression patterns are considered, thus facilitating the search through the whole space of potential gene interactions. Flat profiles are excluded from the analysis, since they are not involved in the observed process in terms of changes in the transcription level.

#### Step2: reverse engineering

##### Dynamic Bayesian Networks

Bayesian Networks are directed acyclic graphs that encode a series of relations of conditional dependence among interacting variables. In the case of gene networks nodes represent the genes and edges represent the relations of conditional dependence among genes. The aim of the learning procedure is to find the network structure G that is most supported by the data D, i.e. that maximize the posterior probability P(G|D). Bayesian Networks do not allow cycles in their topology; therefore, it is not possible to represent feedback control which is actually a critical aspect of gene regulation in real biological systems. To include cycles and feedback control in the regulatory network, Dynamic Bayesian Networks are used, as implemented in the software developed by Kevin Murphy .

##### Reveal

In its original formulation, Reveal uses a Boolean model of the regulation and searches for minimal set of input-genes that can univocally explain the behaviour of the output-gene x from 0–1 discretized data. To explicit possible causative relationships, the algorithm uses the Entropy and Mutual Information score [21] and searches, for each gene x, all the possible interactions of connectivity K = 1. If no genes univocally determines x profile, it searches for all the possible interactions of connectivity K = 2, and if even this search is unsuccessful, it searches in the space of interactions of connectivity K = 3. Reveal stops at connectivity K = 3 for two reasons: first, because search in higher connectivity space is computationally unaffordable; second, when K increases, the disproportion between the number of analyzed genes and the number of available samples causes a more elevated number of false positive discovered relationships [2]. We extended Reveal to data quantized in three levels, with either an instantaneous model of regulation (i.e. y(t) regulates x at time t) or a synchronous one (i.e. y(t) regulates x at time t + Δt).

#### Step 3: sub-networks identification

DBN gives as output a network structure G codified in a connectivity matrix with a non null entry at i^th ^row and j^th ^column representing the relationship found by the algorithm between gene profile j and gene profile i. From the connectivity matrix a network is drawn with genes represented by nodes and regulation by edges, and searched for sub-networks non connected to the remaining of the network. Reveal gives as output a list of input-genes ("regulators") for each "regulated" output-gene. A connectivity matrix is derived from the lists of regulators and sub-networks are identified as for DBN.

### Simulated data

We have developed a simulation tool, able to model concurrent regulation, i.e. a gene affects regulation depending on its interactions with other genes. Synthetic data are generated by simulation of regulatory networks of random scale-free topology, using differential equations in which the rate of change of gene expression is a function of a combination of different regulatory rules.

#### Network topology

Each simulated network consists of H sub-networks. Sub-networks are generated by randomly assigning regulators to each gene, according to a scale-free structure: the probability for each node of having a number of connections with other nodes equal to h is h^-γ ^(γ = 2.2 as observed in [22] for metabolic networks of numerous organisms). The nodes with the highest number of connections are called hubs [23]. Sub-networks are connected to each other through nodes randomly selected among the non hub genes. This strategy gives a scale-invariant characteristic to the simulated network [22].

#### Regulation rules

For a generic gene x, its r regulator genes 1,...,r with expression level y_it_, (i = 1,...,r) at time t, act in concurrency by activating or inhibiting transcription as results of a combination of different rules. The rate of change of gene x expression at time t, depends on the value of the regulatory function R_x_(y_1t_, ..., y_rt_):



where ML_x _is a positive constant, representing the maximum achievable expression value of gene x. The regulatory function R_x_(y_1t_, ..., y_rt_) is a combination of three basic regulatory actions:

(i) min(w_x1_y_1t_, ..., w_xr_y_rt_)/τ processes a regulatory effect achieved only if all the regulators are simultaneously active

(ii) sum(w_x1_y_1t_, ..., w_xr_y_rt_)/τ processes a regulatory action which can be alternatively performed by different regulators

(iii) the minus sign processes a negative regulation (inhibition)

where τ is a time constant and weights w_xi _represent the strength of regulatory action performed by each regulator on the regulated gene x. Complex regulatory actions are obtained by combining the functions described above, e.g. a regulatory function R_x _with five regulators could be R_x_(y_1t_, ..., y_5t_) = min(w_x1_y_1t_, w_x2_y_2t_) - sum(w_x3_y_3t_, min(w_x1_y_1t_, w_x1_y_1t_)). The combination of rules for the regulators and weights w_xi _are randomly chosen, with the only constraint that each gene has at least one activator and one inhibitor.

In order to test the performance of the quantization method, 100 different networks were simulated, each consisting of H = 5 sub-networks and 200 genes. Different topologies and regulatory rules were randomly chosen for each simulation; ML_x _was set equal to 10 for each gene x. To reproduce the data-poor conditions, ten samples were collected from each data set; Gaussian noise with constant standard deviation SD = 0.15 was added to samples ranging from 0 to 10.

Simulated data were quantized in three levels using three different methods: a) the new method based on a model of the experimental error with significance level tuned to reach a compromise between FP and FN classification; b) same as a) but with an arbitrary 5% significance level; c) quantiles based quantization as in [24], i.e. the lowest 33.3% of the values (up to θ_1_) were quantized as -1, the next highest 33.3% (from θ_1 _to θ_2_) as 0, and the highest 33.3% as +1.

Both DBN and Reveal (synchronous model with connectivity K equal to 1) were applied to discrete data.

### Scoring

To assess the performance of the quantization method used in conjunction with the two reverse engineering methods, genes in the identified sub-networks are compared to those in the simulated sub-networks. Precision (number of correctly classified genes among the inferred ones) and Recall (number of correctly classified genes among the true ones) are used at this purpose. More precisely, for each simulated sub-network SIM_h _(h = 1,...,H) and identified sub-network ID_d _(d = 1,...,D), Precision is defined as:



and Recall as:



In order to quantify the ability to identify sub-networks with all or most genes belonging to a single (simulated) sub-network, the maximum Precision across simulated sub-networks SIM_h _(h = 1,...,H) is considered for each identified sub-networks ID_d_. The corresponding Recall is also considered, thus obtaining D pairs of scores (Precision vs Recall) for each simulated data set.

## Results

**Figure 1:**
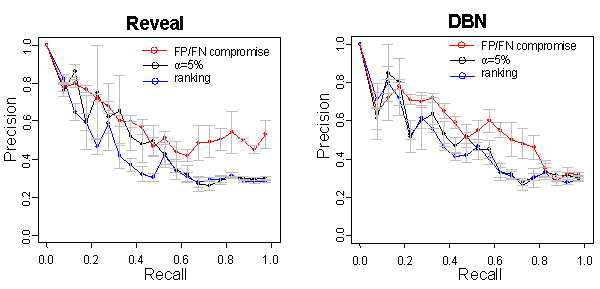
**Precision vs Recall for simulated data. **Average Precision at different Recall intensities obtained on 100 simulated data sets, using Reveal (left panel) and DBN (right panel).

Quantization methods a) and b) require a model of the distribution of the differences between two expression values under the null hypothesis. Since the error is fixed in all simulations as Gaussian with zero mean and a constant SD = 0.15, this distribution is Gaussian with zero mean and a constant SD = 0.15* . Based on this model, the average threshold θ obtained for method a) in 100 simulations equals 0.10 with SD = 0.15. θ in fact varies among 100 simulations, since the significance level α, fixed on the basis of a compromise between FP and FN classifications, depends on N_0_, i.e. the estimated number of samples in the data set that do not change expression with respect to their baseline value (Equations 4–7). The high coefficient of variation of θ indicates that N_0 _strongly depends on the observed dynamics and, thus, on simulated network topology, regulatory rules and initial conditions. Conversely, using quantization method b) the threshold θ is equal to 0.39 for all simulations, according to 5% significance level. For method c) the two thresholds θ1 and θ2 vary among simulations and equal respectively -0.99 with SD = 0.51 and 1.08 with SD = 0.50. Performance of the three methods used with Reveal and DBN are shown in Figure 1 as average Precision at different ranges of Recall intensities (standard error bars are also shown), using Reveal (left panel) and DBN (right panel). For method a) the area under the curve is 0.58 using Reveal and 0.57 using DBN; for method b) it is 0.49 using both Reveal and DBN; for method c) it is 0.43 using Reveal and 0.47 using DBN. These results show that the trade-off between Precision and Recall improves using method a). In particular, for Recalls higher than 40%, Precision obtained using the proposed quantization method a) is consistently higher than that obtained using other methods. Also of interest, the overall performance of Reveal is similar to DBN, in the considered data-poor condition.

## Discussion

A data quantization approach usable with discrete reverse engineering methods has been proposed, which is based on a model of experimental error (known or derived by experimental replicates) and on a compromise between FP and FN classification. Modelling experimental error is a fundamental step since it allows to quantify the error and to assess its distribution. This is particularly important e.g. with Affymetrix chips, since the measurement error is dependent on the expression intensity [25]. In this case, the threshold θ in Equations (1–3), has to be intensity dependent so as to penalize genes expressed at low intensity levels (characterized by high error rates) with respect to genes expressed at high intensity levels (characterized by low error rates) [26]. The quantization method here presented, besides exploiting information on the experimental error, derives θ on the basis of the variability of the data-set to be discretized. In fact θ corresponds to a significance level α chosen so as to compromise between FP and FN classifications, where FP and FN are estimated on data, based on the number N_0 _of samples that do not change expression with respect to their baseline value (Eq. 4–7). The other two quantization methods we have considered are based respectively on a model of the experimental error, but with a threshold corresponding to a 5% significance level, and on rank sorting. The first takes into account the experimental error, but with an arbitrary threshold level independent from the data-set variability, the second does not exploit information on experimental error, but, by using ranking, takes somehow into account the data-set dispersion.

**Figure 2:**
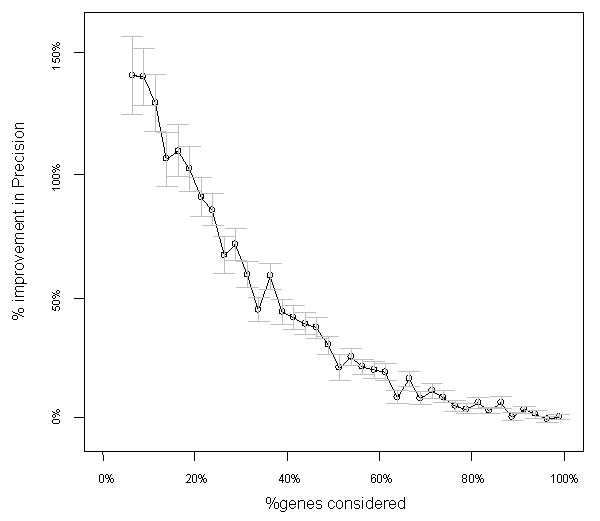
**Improvement in Precision obtained using ranking. **Per cent improvement in Precision obtained ranking genes and considering increasing percentage of them as belonging to identified modules.

To quantify the performance of the quantization methods when used with reverse engineering, synthetic gene expression profiles were generated from completely connected scale-free networks of 200 genes; 10 time samples were collected from each gene profile to reproduce the data-poor situation. Application on 100 synthetic data sets indicated that: 1) quantization based on compromise between FP and FN classifications improves the algorithm performance; 2) Reveal and DBN perform similarly. Figure 1 shows the trade off between Precision and Recall for the identified sub-networks in the 100 simulations. It is of interest to concentrate on Precision, which is related to the false positive rate in predicted sub-networks. Precision can be improved by focusing on genes that could be central in the regulation (possible hubs). To this purpose, each identified sub-network was searched in order to rank nodes on the basis of the degree of connectivity (i.e. number of connections with other nodes). The gene with highest degree was ranked first; other nodes were ordered depending on their degree of connectivity, with the constraint of having at least a direct connection with the genes previously ranked. Figure 2 shows the average per cent improvement in Precision (standard error bars are also shown) obtained by applying the ranking step to Reveal results (quantization performed with method a).

Improvement in Precision depends on the percentage of genes considered from the ranking. It is above 50% when less than 40% of the ranked genes are considered and still reasonable, above 25%, with less than 60% of the genes. Precision thus substantially improves with ranking, but Recall obviously deteriorates. Back to results of Figure 1, if Recall is higher than 40%, Precision ranges between 40% and 60%. This limited range of Precision may have several explanations. First, the model implemented in Reveal and DBN to find cause-effect relationships is much simpler than the model used to generate simulated data and may not distinguish behaviours of different complexity. The simulation model is in fact based on differential equations in which the rate of change of gene expression is a function of a combination of different positive/negative regulatory actions, e.g. achieved only if all the regulator are active simultaneously or alternatively performed by different regulators. In this sense the simulation model used in this paper combines characteristics of models based on differential equations [24,27] as well as on Boolean networks [28]. Boolean networks describe important aspects of gene regulation such as complex concurrent regulatory mechanisms, but do not describe continuous changes in gene expression. In contrast, differential equation based models generate continuous data and allow to include the processes of transcription and mRNA degradation, but, in general, do not address regulatory logic more complex than additive or multiplicative effects. The strategy we adopted combines the major advantages of the two approaches.

A second source of FP may arise from the use of discrete data, which is a simplified representation of gene expression. To assess to which extent the use of discrete rather than continuous data is critical, we compared our results on simulated data with those obtained by applying a continuous reverse engineering method such as ARACNe [16]. ARACNe uses an extension of Mutual Information to continuous data [29] and, as other continuous methods, explicitly requires hundreds of data points to perform the analysis to a sufficient degree of accuracy and was not proposed to address sparse datasets. However, at variance with other continuous methods, it does not require model parameter identification and is computationally affordable. Therefore, we explored its use on simulated data sets for sake of comparing discrete vs continuous approaches in data-poor conditions. When ARACNe was used to identify subnetworks on simulated data (5 sub-networks for each simulated data set), it always identified a single sub-network with Recall ranging from 0.8 to 1 and Precision always equal to 0.2, thus indicating random results; when ARACNe was used to reconstruct the entire regulatory network, Recall and Precision ranged between 0.2–0.8 and 0–0.03, respectively. These results confirm that the use of discrete rather than continuous data is advantageous when few samples are available. Continuous approaches are likely to become advantageous with increasing number of samples.

## Conclusion

A new method was presented to quantize data in a statistically robust way, which can be used as a preliminary step to discrete reverse engineering algorithms. The performance of the method was tested with two basic discrete reverse engineering methods: Reveal and Dynamic Bayesian Networks, using continuous synthetic data generated by a simulation of regulatory networks of random scale-free topology. The simulation model generates continuous data using differential equations and uses an extension of Boolean logic to continuous data to mimic regulatory programs. The new quantization method improves Precision and Recall trade-off, both with Reveal and DBN. Reveal and DBN perform similarly on simulated data.

## Authors' contributions

BDC and FSC conceived the study and performed data analysis under the guidance and supervision of GT. BDC designed and implemented the current version of the algorithm and of the simulation model here presented. SKN, ZT and CC were responsible for the overall conception and project coordination. All authors read and approved the final manuscript.

## Appendix

By assuming a log-additive error model as in [30], the log-expression of a generic gene x in replicates a and b, can be expressed as:



where μ represents the actual (unknown) gene expression and ε_a_, ε_b _two realizations of the error. To quantify the difference between two expression values under the null assumption, a variable δ is defined as:

δ = log(x_a_) - log(x_b_) = ε_a _- ε_b _    (A2)

Different distribution models (t-Student distribution, bi-exponential distribution, and mixture models of N Gaussians, N = 1, ..., 6) can be used to fit the entire set of δ values obtained by applying Equation (A2) to all genes and available replicates. Once the best model is selected based on a number of criteria, θ is evaluated from the distribution of δ according to a significance level α.
